# A task scheduling algorithm with deadline constraints for distributed clouds in smart cities

**DOI:** 10.7717/peerj-cs.1346

**Published:** 2023-04-14

**Authors:** Jincheng Zhou, Bo Liu, Jian Gao

**Affiliations:** 1 School of Computer and Information, Qiannan Normal University for Nationalities, Duyun, Guizhou, China; 2 Key Laboratory of Complex Systems and Intelligent Optimization of Guizhou Province, Duyun, Guizhou, China; 3 State Key Laboratory of Public Big Data, College of Computer Science and Technology, Guizhou University, Guiyang, Guizhou, China; 4 College of Information Science and Technology, Northeast Normal University, Changchun, Jilin, China

**Keywords:** Smart cities, Task scheduling, Distributed clouds, Local search algorithm

## Abstract

Computing technologies and 5G are helpful for the development of smart cities. Cloud computing has become an essential smart city technology. With artificial intelligence technologies, it can be used to integrate data from various devices, such as sensors and cameras, over the network in a smart city for management of the infrastructure and processing of Internet of Things (IoT) data. Cloud computing platforms provide services to users. Task scheduling in the cloud environment is an important technology to shorten computing time and reduce user cost, and thus has many important applications. Recently, a hierarchical distributed cloud service network model for the smart city has been proposed where distributed (micro) clouds, and core clouds are considered to achieve a better network architecture. Task scheduling in the model has attracted many researchers. In this article, we study a task scheduling problem with deadline constraints in the distributed cloud model and aim to reduce the communication network’s data load and provide low-latency services from the cloud server in the local area, hence promoting the efficiency of cloud computing services for local users. To solve the task scheduling problem efficiently, we present an efficient local search algorithm to solve the problem. In the algorithm, a greedy search strategy is proposed to improve the current solutions iteratively. Moreover, randomized methods are used in selecting tasks and virtual machines for reassigning tasks. We carried out extensive computational experiments to evaluate the performance of our algorithm and compared experimental results with Swarm-based approaches, such as GA and PSO. The comparative results show that the proposed local search algorithm performs better than the comparative algorithms on the task scheduling problem.

## Introduction

In recent years, there has been a growing interest in the technology of smart cities to settle the issues of population growth and urbanization. In a smart city, technology and data are employed to increase the efficiency of services and reduce costs, thus achieving a better life for citizens. The technology in smart cities includes IoT devices, data analytics, and machine learning for city management, such as optimizing traffic flow and resource allocation (Belanche, Casaló & Orús, 2016; Yigitcanlar et al., 2018). As the resources, environment and infrastructure of a city are digitized, they can be used to collect and analyze data from sources, such as IoT devices and sensors, to achieve intelligent management and improve management level (Su, Li & Fu, 2011).

Intelligent devices and wireless sensors provide a technical means to achieve intelligent transportation, smart home, health monitoring, environmental detection, and other scenarios (Gaur et al., 2015; Arasteh et al., 2016; Xia et al., 2013; Yao et al., 2013). A smart city requires a large number of smart devices. Through the Internet, it can share data with other devices, and 5G network technology ensures fast data transmission, low latency, and better connectivity (Rao & Prasad, 2018). Moreover, cloud computing plays an essential role in enabling the implementation of smart city. It is used to manage these intelligent devices and analyze data. By collecting and analyzing data in the city, computing systems can offer an extensive range of computing services and thus help to make decisions for the operation of smart cities through cloud computing and communication technologies.

In smart cities, solving scheduling problems reasonably and efficiently can promote service levels in various scenarios (Wu et al., 2019; Vigneswari & Mohamed, 2014; Gao et al., 2018). In a cloud environment, we shall assign tasks to virtual machines and execute them, so how to allocate computing resources appropriately is a key technology in cloud computing to improve the efficiency of cloud systems (Zheng, Li & Guo, 2021). Therefore, resource allocation and task scheduling were studied in the cloud systems to ensure the performance of cloud services and the requirements of users. The purpose of task scheduling in the cloud environment is to allocate tasks to appropriate machines so as to improve resource utilization, shorten computing time, and reduce costs. Usually, solving task scheduling problems in cloud environments is not easy, as most of the problems are hard computational problems (Kalra & Singh, 2015; Gökalp, 2021). Due to the NP-hardness, finding the optimal solutions becomes impossible when the scale of the problem grows too large. Therefore, heuristics and meta-heuristics are usually used to find sub-optimal solutions within a reasonable time. There are a large number of heuristic and meta-heuristic methods for the various task scheduling problems.

Tawfeek et al. (2013) proposed a cloud task scheduling algorithm based on an ant colony optimization. The algorithm allocates tasks in the cloud system to shorten the task completion time. NZanywayingoma & Yang (2017) proposed an improved particle swarm optimization, which can allocate dynamic virtual resources and reduce the total time of task scheduling in the cloud environment. Nie, Pan & Wu (2020) improved the ability of the traditional ant colony algorithm to shorten the task completion time. It searches for the global optimal solution by increasing the load balance adaptive factor and enabling tasks to be assigned to the most appropriate cloud virtual machine. To minimize the completion time, Nabi et al. (2022) introduced a particle swarm optimization algorithm with linear descent and adaptive inertia weight strategies; Manikandan, Gobalakrishnan & Pradeep (2022) proposed a new hybrid algorithm to solve a multi-objective task scheduling problems in cloud computing environments; Bezdan et al. (2022) proposed a hybrid bat algorithm to minimize the cost and completion time.

Moreover, deadline constraints are also considered in the field of task scheduling in cloud environments. Zuo, Zhang & Tan (2013) studied a task scheduling problem with deadline constraints, and proposed a particle swarm optimization algorithm with an adaptive update strategy. Verma & Kaushal (2014) used a heuristic genetic algorithm to solve the task scheduling problem with deadline constraints and use the priority of tasks to minimize execution costs. Dubey & Sharma (2021) used a hybrid task scheduling algorithm to optimize multi-objectives under deadline constraints.

Besides, local search algorithms play an essential role in task scheduling. Local search is one of the commonly used heuristic methods for combinatorial optimization problems (Aarts & Lenstra, 2003). It has been proved that local search is a simple and effective method that can solve many computational problems, from computer science and mathematics to engineering and bioinformatics. As an important method in heuristic optimization methods, local search methods are also used to deal with task scheduling problems and show good performance (Wu, Shu & Gu, 2001).

On the one hand, local search is incorporated into meta-heuristics. For example, Kumar, Kousalya & Vishnuppriya (2017) used local search in the discrete symbiotic organism search to achieve better performance. On the other hand, local search was employed as the main framework of the task scheduling algorithms. Estellon, Gardi & Nouioua (2009) used local search to solve the resource allocation problem in reality with good performance and efficiency. Zhang, Wang & Yuan (2018) used iterative local search to ensure the performance of multi-objective task scheduling. In the application of a cloud system, Loheswaran, Daniya & Karthick (2019) improved the utilization of cloud computing resources based on local search. Qin, Pi & Shao (2022) proposed an iterated local search algorithm in the cloud systems for workflow scheduling. Xing et al. (2022) used local search to solve a workflow scheduling problem with random task runtime.

In recent years, distributed cloud systems have become an increasingly popular trend. Li (2020) proposed a layered distributed cloud service network model for smart cities. In the model, a framework of distributed cloud systems with micro clouds is studied to achieve low latency. Li et al. (2022) proposed an optimal data deployment algorithm to reduce data access cost and data deployment time in distributed cloud environments. Distributed clouds allow for distributed computing resources to be used in different locations. Those systems can increase scalability, reliability, and security, and also enhance the ability of lower latency to users in a local area. For example, in a distributed academic cloud system, each organization has its own local cloud and the cloud is connected with other organization’s clouds and public clouds. The users always hope to perform their jobs in the local clouds to achieve low latency and high security, but if there is no sufficient computing resources in the local clouds, jobs have to be allocated to public core clouds or other organization’s clouds. Therefore, task scheduling algorithms for such distributed clouds are beneficial in practice when the computing infrastructures are spread across multiple vendors and locations.

Although we have viewed many algorithms for task scheduling problems in cloud computing, there are relatively few algorithms to schedule tasks in distributed clouds. In this article, we study a task scheduling problem with deadline constraints in the distributed micro-cloud environment. In the problem, computing resources are spread out across multiple locations rather than in a single data center, so communication latency can be lowered by assigning tasks to their users or data. To achieve low latency and high performance, we propose an efficient local search algorithm based on greedy and perturbation strategies to solve the problem. Different from existing task scheduling algorithms that are mainly based on evolutionary algorithms, our method is a simple but effective local search approach. It first constructs an initial solution and improves it iteratively. The search strategies in the local search algorithm are critical to improve convergence speed and solution quality. To solve the problem effectively, we introduced a greedy search strategy for finding better solutions; To avoid trapping in a local optimum, we incorporated a perturbation strategy into the algorithm, so that solution diversity is enhanced. To further enhance the diversity of solutions, we employed a randomized search method, which triggers perturbation with a probability mechanism. In the local search, the greedy strategy and the perturbation strategy are performed alternatively. Both are the key components of our algorithm. Moreover, we carried out extensive experiments to test our approach. We analyzed the comparative results of experiments intensively and showed that the proposed algorithm is able to produce much better solutions than evolutionary algorithms. Computational experimental results show that the algorithm outperforms other algorithms. Both the rejection rate and the total cost of running the tasks are better than the results yielded by comparative algorithms. We also analyzed the effectiveness of our strategy in the algorithm.

The remainder of this article is organized as follows. The next section introduces the task scheduling models and notations used in the article. Section 3 presents our proposed local search algorithm and the strategies employed in the algorithm. “Simulation and Performance Evaluation” analyzes the comparative experimental results. Finally, we give some conclusions.

## Problem description

In this section, we describe the task scheduling problems in the smart city environment in this article, and explain tasks, machines and the cost for processing tasks in the problem.

First, there is a set of physical machines (servers). The physical machines belong to different clouds including local micro-clouds and core-clouds. The cloud systems are distributed in different locations in a city. We denote the machines set as $P = \left\{ {{p_1},...,{p_m}} \right\}$, where *m* is the number of total machines in the distributed cloud system of the city. The computing capacity of physical machines is heterogeneous, and Million Instructions Per Second (MIPS) is used to measure the capacity. The capacity of machine ${p_i}$ is denoted by $MIP{S_i}$.

Then, we introduce the task model. There are *n* independent tasks in the problem denoted by $T = \{ {t_1},{t_2},...,{t_n}\}$. Each task *t*_*i*_ is associated with a deadline *d*_*i*_ that restricts the latest completing time of the task, and also associated with a machine set ${P_{{t_i}}} \subseteq P$, which is the machines in the local area of ${t_i}$. The task has a length of workload *l*_*i*_, an input data size *in*_*i*_ and an output data size *out*_*i*_. Since a task belongs to a user and thus has a local cloud, the task can achieve fast data transmission and a low latency if it is processed by a machine in the local micro-cloud. Moreover, we assume all the tasks in *T* are ready for processing at the beginning. The tasks are non-preemptive in nature. In the case of non-preemptive scheduling cannot interrupt in the middle of the execution. So when a task is under processing, the tasks cannot be transferred to other machines and other tasks assigned to the same physical machine should be waiting until the task completes. To solve the scheduling problem, we should map virtual machines for processing tasks onto physical machines.

Since the physical machines belong to different micro-clouds, the transmission speed differs in each machine for each task. For example, a task can upload its data to a machine in the local network in a short time because of the high bandwidth in the local network and information can be sent quickly, but the transmission may suffer from a high delay when it communicates with a remote machine. Therefore, in our model, transmission speeds are different for each task to each machine. Data security is another important issue in cloud computing. Some private data must be transferred in a confidential way, security techniques, such as DES cryptographic algorithm and MD5 hash algorithm, can be used to ensure a certain level of security, but performing those encryption algorithms require extra overhead. Therefore, users always prefer to process tasks on their local clouds, and achieve low latency and high security.

Processing time is also considered in the problem. To solve the problem, we shall assign a task to a machine and assign a start time to it. Thus, the completion time of processing the task can be computed by adding the execution time to the start time. Moreover, the problem model includes the deadline constraints that restrict the time a task must be completed. To that end, we should satisfy the deadline constraints, so the completion time of all the tasks should be smaller than or equal to their corresponding deadlines. Note that some problem instances may not have a feasible solution such that all the tasks satisfy the deadline constraints. State in another way, not all the tasks can be assigned to an appropriate machine such that they can complete within the deadline, and some tasks have to be rejected without an assignment to a certain machine. In this case, we hope the cloud system can process as many tasks as possible. Formally, we can calculate processing time and completion time as follows.

The processing time is composed of execution time and transmission time. The execution time of task *t*_*i*_ on machine *p*_*j*_ is $\; {l_i}/MIP{S_j}$, where *l*_*i*_ is the length of workload of the task *t*_*i*_ and *MIPS*_*j*_ is the million instructions per second of machine *p*_*j*_. The transmission time is $\left( {i{n_i} + ou{t_i}} \right)/{b_{ij}}$, where *b*_*ij*_ is the bandwidth of machine *p*_*j*_ for task *t*_*i*_. Then, we can obtain the processing time as:

(1) $$tim{e_{ij}}\; = \; {l_i}/MIP{S_j} + \left( {i{n_i} + ou{t_i}} \right)/{b_{ij}}$$

Given a machine *p*_*j*_ a start time *s*_*i*_ of task *t*_*i*_. Then, we can compute its complete time as

(2) $${e_i} = {s_i} + tim{e_{ij}}.$$

In the following, we introduce the cost model. The total cost of all the tasks is a goal of the scheduling problem. The cost of processing a task on a machine depends on the processing time, and the fee of using a machine is charged in $ per hour. As the computing capacity of a machine is differ from others. Usually, a higher price of unit running time means a more powerful computing capacity, and therefore the fees for the machines are different. Suppose task *t*_*i*_ is assigned to the machine ${p_j}$ with start time ${s_i}$, then the cost of processing is ${\rm \; C_{\it i}} = {\rm time_{\it ij}} \times fe{e_j}$, where $fe{e_j}$ is cost per hour on machine ${p_j}$.

The aim of solving the task scheduling problem is to find a solution (an assignment of tasks to virtual machines and start times for tasks) such that one task is assigned to exactly one machine if it can satisfy the deadline constraint or it is rejected by the cloud system. For each task ${t_i}$, we have the constraint:

(3) $${e_{i\; }} \le {d_i}.$$

Three objectives are optimized in the problem. Formally, the objectives are expressed as:

(a) maximizing the number of tasks completed within deadlines.

(4) $$maximize\; \left| {{T_d}} \right|,\; {\rm where}\;{T_d}\; = \; \{ {t_i}|\; {e_i} < {d_i}\}$$

(b) maximizing the number of tasks assigned to their local clouds.

(5) $$maximize\; \left| {{T_l}} \right| \; {\rm where}\;{T_l} = \{ {t_i}|\; {t_i}\; is\; {\it assigned}\;\rm to\; a\; machine\; in\; {P_{{t_i}}}\}.$$

(c) minimizing the total cost of processing all the tasks.

(6) $$minimize\; C\; = \; \mathop \sum \nolimits_{i \in \left\{ {1,...,n} \right\}} {C_i}$$

where $C$ is the total cost for executing all the tasks.

In the problem, we give priority to maximizing the first objective $\left| {{T_d}} \right|$, and then the second one $\left| {{T_l}} \right|$; if the two goals are the same, we try to optimize the total cost $C$. This is because the cloud system should process tasks within their deadlines as many as possible, and then tasks should be processed in their local clouds as many as possible; at last, it minimizes the total cost $C$ of processing all the tasks.

It is easy to see that the task scheduling problem in the distributed cloud environment is NP-hard as it is reported that single-machine scheduling with deadlines is NP-hard (Sahni, 1976).

## Proposed local search algorithm

### Local search

First, we briefly introduce heuristics and local search techniques for solving constraint optimization problems, and then present our local search-based algorithm in the following subsection. Heuristic algorithms are important tools for solving constraint optimization problems, because they can produce satisfactory solutions in a reasonable time. Local search is one of the heuristic methods for combinatorial optimization problems in computer science and artificial intelligence. It has been demonstrated that local search is a simple but effective method for solving numerous computationally hard problems in computer science, mathematics, engineering, and bioinformatics, including the maximum satisfiability problem (Gao, Li & Yin, 2017; Luo et al., 2017, 2014), timetable scheduling (Psarra & Apostolou, 2019), and clustering (Tran et al., 2021; Levinkov, Kirillov & Andres, 2017). To solve complex optimization problems, local search algorithms with various search strategies have proven very effective in the literature. Typically, a local search algorithm begins with a randomly generated starting solution and then looks for a better solution by traversing the candidate solution space. Various local change techniques have been developed for exploiting solution space in local search algorithms. When a certain number of rounds have been performed or a predetermined amount of time has passed, the algorithm terminates and returns the best solution it found. In contrast to evolutionary-based algorithms that construct a population and iteratively improve individuals, the local search progressively improves a single solution. It always works well and finds an approximation of the optimal solution to the problem.

### Solution representation and initialization

As a local search algorithm starts with an initial solution, we introduce the structure of a solution and its initialization. As the aim of the problem is to assign tasks to machines, a task sequence is required for each machine, and then with an order of tasks we can compute their start times accordingly. Hence, task sequences have to be defined in the solution. Moreover, it is clear that not all the tasks can be assigned to a machine if the deadline constraints of the tasks are too tight, because some tasks may violate the constraints whenever they are assigned to any machine. In this case, we introduce a conflict task set to store the tasks that fail to satisfy the deadline constraints. As a result, in a solution, a conflict task set and *m* task sequences are defined.

We provide an example to demonstrate the solution structure. Suppose there are 10 tasks and three machines, and then a solution is composed of three sequences and a conflict task set. For instance, the solution $\{ \{ {t_5},{t_2},{t_4}\} ,\{ {t_9},{t_3},{t_6}\} ,\{ {t_1},{t_8}\} ,\{ {t_7},{t_{10}}\} \}$ strands for machine *p*_1_ processes *t*_5_, *t*_2_, *t*_4_, *p*_2_ processes ${t_9},\;{t_3},\;{t_6}$, and *p*_3_ processes *t*_1_,*t*_8_, while the *t*_7_ and *t*_10_ are in the conflict set.

In the initialization, we use a simple and random way to initialize the solution. To be specific, for each task, it selects a random machine and assigns it to the machine. After all the tasks are assigned to machines, the tasks are ordered by their deadlines to form a task sequence, and then we can compute the total cost for a certain machine and the number of rejected tasks. There are two cases here that should be considered. The first one is that all the tasks assigned to the machine do not violate the deadline constraints, that is all of them are scheduled to finish their processing before the deadlines, and this is a legal assignment, so the construction method will accept the assignment. The second one is that there exist some tasks whose completion times are behind their deadlines. If so, such tasks should be removed from the task sequence of the machine. Note that a remove of a conflict task may result in its subsequent tasks satisfying the deadline constraints, so the cost and the rejected tasks are recomputed. The remove of conflict tasks and recomputed are performed alternatively until there are no conflict tasks. All the removed tasks are put into the conflict task set. Therefore, we construct a random initial solution.

### Proposed algorithm

In this subsection, we present the main framework of our proposed local search algorithm, and leave the detailed introduction of components in the following subsection.

Algorithm 1 indicates the procedure in detail. It first constructs a solution as the initial one of the local search. The construction is a random approach as mentioned above. In the initialization, the algorithm also initializes a counter, denoted by *noimpr*, that counts the number of non-improving steps. Afterwards, the local search algorithm is executed iteratively, where a greedy strategy (we will introduce them in detail in the following subsection) and a perturbation method are adopted. Two strategies are performed alternately in the iterated local search procedure. The greedy strategy aims at improving the solutions in terms of the number of conflict tasks that violate the deadline restrictions ( $n - \left| {{T_d}} \right|$ in solution *S*), denoted by $conflict\left( S \right)$, and the cost of all the tasks with the penalty of tasks assigned to non-local micro clouds, denoted by $obj\left( S \right)$ (we will define the function formally in the next subsection). To maintain solution diversity during the search, randomized strategies are often adopted, and a random move strategy is always integrated into the algorithm to avoid trapping in local optima. In our algorithm, we employ perturbations on the local optimal solution obtained by local search, and then exploit the neighborhood iteratively. When the greedy method cannot improve the current solution for a certain rounds, *i.e*., $noimpr$ reaches the threshold $thr$ (we set the threshold $thr = 5 \times n \times p$ in our experiment), the algorithm performs a perturbation step to escape the local area, because the current solution is a local minimal solution. Therefore, random assignments for several randomly selected tasks are done. In addition, when the termination condition is satisfied, the algorithm stops and returns the best found solution.

**Algorithm 1:** Local search algorithm (LS)

1 initialization and construct an initial solution randomly;
*2 noimpr ←* 0; let *S*_*best*_ be the initial solution;
3 **While** not reach the limited time **do**
4 choose a task *t* from the task list randomly;
5 * S ← greedy_strategy*(*S,t*);
6 * ***If** *t* is not moved **then**
7 * noimpr ← noimpr +1*;
8 * ***Else**
9 ** ***noimpr ←* 0;
10 * ***If** *noimpr > thr or random() < thp* **then**
11 * ***If** *conflict(S*_*best*_*) < conflict(S) or (conflict(S*_*best*_*) = conflict(S) and obj(S*_*best*_*) < obj(S))* **then**
12 * S*_*best*_ *← S*;
13 choose *r* tasks from *T*, and move them to random machines;
14 *noimpr ←* 0;
15 **Return** *S*_*best*;_

We devise a randomized method and incorporate the method into our algorithm to enhance the diversity of the local search ability. Note that despite the integration of diversity strategies in the task and machine selection, the algorithm can still be trapped in a local area if no improvement can be made by moving any node. This is because the algorithm repeats the selection and tries to find a better place for the selected tasks, and the method clearly leads to a local optimum after a series of better moves.

To escape local optima, we combine the algorithm with a probability-based perturbation. The strategy is devised to make a perturbation when the algorithm achieves a local best solution. The perturbation method chooses a task from *T* and moves it to a randomly selected machine. The move will be repeated *r* times so *r* tasks will be moved (we set $r = \left\lceil {n/10} \right\rceil$ in our experiment).

To further enhance the search diversity, we add a probability in the trigger condition of the perturbation. The threshold *thp* is a pre-defined parameter. With the probability *thp* the local search algorithm triggers the perturbation step regardless whether the counter *noimpr* reaches the threshold *thr*. The function *random()* returns a real number between 0 and 1.

### Components of local search

In this subsection, we introduce the greedy strategy used in the proposed algorithm.

The greedy strategy is a critical component in our algorithm. It determines how to change and improve the solution. The objective function to be optimized is essential for the greedy strategy. Many algorithms mix the objective function and penalty of violated constraints together, and thus a hybrid objective function is usually defined to compare solutions. In such methods, the constraint optimization problem is converted into an unconstrained optimization problem. However, this method may fail to handle hard constraints, because a mixed function guides the simultaneous optimization of the number of violated constraints and the objective function in the search algorithm. This usually leads to an infeasible solution in which the constraints cannot be satisfied, and sometimes decreases the convergence speed.

Different from existing methods, in our algorithm, we treat the number of violated deadline constraints and the cost separately. To this end, we consider deadline constraints as hard constraints, and the number of tasks in local micro clouds and the total cost *C* are treated as the goal to be optimized. Therefore, to assign tasks to local clouds as many as possible, we add a penalty to the cost function by multiplying a parameter *α* to the cost whose tasks are assigned to non-local machines. The parameter *α* is a real number above 1.0. Thereafter, we define the objective function:

(7) $$minimize\; obj\left( S \right)\; = \mathop \sum \nolimits_{i \in \left\{ {1,...,n} \right\}} {{\rm {\mathbb C}}_i}\;$$

where

$\eqalign{{{\rm {\mathbb C}}_i} = \left\{ {\matrix{ {{\hskip-40pt C_i} \quad if\; {t_i}\; \in \; {T_l}} \cr { {\hskip-3pt \alpha {C_i} \quad otherwise\; \left( {{t_i}\; \notin \; {T_l}} \right). } }} } \right.}$

Based on the above discussion, our algorithm should find a solution that violates as few deadline constraints as possible because constraint satisfaction is the prime goal and minimizing the function *obj* as the secondary goal.

Algorithm 2 shows the detailed procedure of the greedy strategy. In the algorithm, a task is selected randomly. Then, we try to find a new machine and assign the task to it, if the new assignment can obtain a better solution. For each machine (the machine is selected in random order), we calculate the solution after moving task *t*, and compare it with the current solution. If a better solution is found, we stop trying other machines. If assigning to the machine cannot improve the current solution, it will choose another machine for assigning the task until a certain number of attempts have been performed. After these failed attempts, we suppose there does not exist a better place for the task, so it will not be ignored and a new task is selected for the next round of attempts. The function *move*(*S*,*t*,*p*) is defined to insert the task *t* to the task sequence of the new machine *p*; To be specific, it tries to insert all the possible positions in the task sequence of *p*. It inserts the task to the position before the first task whose deadline is bigger than *t*’s deadline or the last position if there is no such task. It returns the new solution after the move.

**Algorithm 2:** *greedy_strategy*(*S,t*)

1 **Foreach** machine *p* **do**
2 **If** *t* is assigned to *p* **then** continue;
3 *S’ ← move(S,t,p)*;
4 **If** *conflict(S’) < conflict(S)* **then**
5 *S ← S’*; *break;*
6 **Elseif** *conflict(S’) = conflict(S)* **then**
7 **If** *obj(S’) < obj(S)* **then**
8 *S ← S’*; *break;*
9 **Return** *S*;

Besides the machines in the problem, we define a task set to store rejected tasks, that is, the tasks that cannot be inserted into a sequence of a machine due to the restriction of the deadline constraints. Note that if there is a conflict task (the task is completed behind its deadline) after adding *t* to *p*, *t* is not inserted into *p* in the new solution and it is added to the rejected task set instead. The function *move*(*S,t,p)* is executed to move task *t*, and if the insertion of *t* to the sequence of machine *p* leads to some tasks violating the deadline constraints, these tasks will be removed from the task sequence and put into the rejected task set.

In the following, we analyze the time complexity of the greedy strategy. In the strategy, the time of computing conflicts and *obj* is *O*(*n*), and at most it has to try *m* machines; Also, the function move () is used to change the position of *t*, and task move can be done in *O*(*n*). Therefore, the time complexity of the function *greedy_strategy* is *O*(*nm*).

## Simulation and performance evaluation

In this section, we conduct extensive experiments and analyze the results to evaluate our proposed algorithm. Also, we compare our algorithm with GA and PSO, which are important algorithms in task scheduling.

### Experimental setup

Our algorithm was implemented with Java language, and compiled and run it with JDK 1.8. We take the algorithms GA and PSO proposed as baselines. We generate task scheduling instances with the task number ranging from 100 to 300, and the number of machines is 5 to 20, and 45 instances with nine groups are tested in our experiment. For a fair comparison, we run our algorithm and the other comparative algorithms 10 times for each task scheduling instance. The time limitation is set to 30 s because the algorithms sometimes fail to solve the instances in a reasonable time. We run all the algorithms on a computer with an Intel(R) Core(TM) i7-11700 CPU (2.50 Hz) and 16 GB RAM running Windows 10. We calculate the average results and the best results of 10 runs to evaluate algorithm performance.

Genetic Algorithm (GA) is a meta-heuristic algorithm that searches for optimal solutions by simulating the laws of biological evolution in nature. It transforms the problem-solving process into operations such as random selection, crossover, and mutation of the population. After continuous evolution and elimination from generation to generation, it finally converges into a group of optimal individuals (that is, optimal or near-optimal solutions) that adapt to the environment. Genetic algorithm has the advantages of high efficiency, parallel, strong global search ability, and strong scalability, easy to combine with other algorithms, so it is the most widely used. Moreover, it is also very popular to solve various task scheduling problems (Verma & Kaushal, 2014; Abdullahi et al., 2019; Houssein et al., 2021).

Particle swarm algorithm (PSO) is a meta-heuristic algorithm that simulates the foraging behavior of flocks of birds, the task of a flock of birds is to find the largest food source (global optimal solution) in the search space. The solution to every optimization problem is a bird in the search space, called a particle. In each iteration, the particles transmit their position information and optimal solution information to each other during the search process, and find the global optimal solution by following the optimal value searched by the current individual and the optimal value of the population. Because of its good ability to solve combinatorial optimization problems, it is a good algorithm for task scheduling, and we have viewed many works of PSO-based scheduling approaches (Houssein et al., 2021; Zuo, Zhang & Tan, 2013; Dubey & Sharma, 2021; Nabi et al., 2022) in the last decade.

### Comparisons with existing algorithms

In this subsection, we test our algorithm LS and analyze comparative results with existing evolutionary-based algorithms, which have been used to solve various task scheduling problems recently. We evaluate the number of rejected tasks, the number of tasks assigned to local machines and the total cost. As we hope to process as many tasks as possible, for an instance, we first check the number of rejected tasks, and the fewer rejected tasks mean the solution is better. If the rejected task numbers are equal in the two solutions, we compare the number of tasks that are assigned to local machines, and then compare the total cost. Table 1 gives the detailed results of all the 45 instances, where the average number of rejected tasks, the average number of local tasks, and the average cost for each instance over 10 runs are listed.

**Table 1:** Comparative results of the average number of rejected tasks and average cost.

	LS			GA			PSO		
Instance	#Rejected	#Local	Cost	#Rejected	#Local	Cost	#Rejected	#Local	Cost
100-5-1	0	96	3,724.551	0	54.9	3,765.430	0.1	39.3	3,779.640
100-5-2	0	96.3	3,894.464	0	58.1	4,023.193	0	44.2	4,022.386
100-5-3	0	96	3,714.779	0	53.2	3,761.939	0.2	35.3	3,811.037
100-5-4	0	95.6	4,308.530	0	54.1	4,320.342	0.5	37.1	4,318.105
100-5-5	0	97.1	3,761.247	0	54	3,890.961	0	36.3	3,876.139
100-10-1	0	82.9	3,436.600	0	33.5	3,500.484	2.2	20.6	3,482.373
100-10-2	0	83.5	4,151.194	0	34.8	4,246.553	1.5	19.7	4,246.219
100-10-3	0	79.3	3,866.267	0	31.3	3,947.463	2.5	20.6	3,925.971
100-10-4	0	85.1	4,098.065	0	32.3	4,112.542	2.5	20.4	4,138.753
100-10-5	0	69.9	3,676.510	1.1	20.2	3,696.339	5.8	18.1	3,696.068
150-5-1	0	144.6	5,585.126	0	80.4	5,684.004	0	60.2	5,693.055
150-5-2	0	145.9	5,874.865	0	78.1	6,017.318	0	64.5	6,090.887
150-5-3	0	144.1	5,723.080	0	78.5	5,885.210	0	60.1	5,919.457
150-5-4	0	145.5	6,194.204	0	72.7	6,222.323	0.6	52.4	6,184.208
150-5-5	0	146.8	5,430.327	0	77	5,634.843	0	57.8	5,617.953
150-10-1	0	126.6	5,326.577	0	44.1	5,379.713	2.5	30.2	5,427.539
150-10-2	0	125.4	6,266.869	0	47.8	6,416.550	2.1	33.8	6,422.384
150-10-3	0	127.6	5,664.869	0	46.5	5,856.876	1.6	30	5,883.811
150-10-4	0	131.4	6,279.622	0	46.6	6,330.217	2.4	29.6	6,317.944
150-10-5	0	130.4	5,373.756	0	47.9	5,436.634	1.9	28.9	5,487.804
150-15-1	0	112.9	5,049.841	0	37.2	5,171.035	5.3	29	5,174.500
150-15-2	0.3	94.6	5,607.956	3.7	35	5,674.369	11.1	32.8	5,665.062
150-15-3	0	116.2	5,745.012	0	38.2	5,841.514	3.9	26.6	5,832.087
150-15-4	0	113.3	6,161.661	0.9	29.9	6,198.171	8	30.5	6,208.942
150-15-5	0	113.1	5,797.540	1	31.1	5,831.609	7.5	30.3	5,866.051
200-10-1	0	170.9	6,883.605	0	61.8	7,028.413	1.2	44.6	7,063.412
200-10-2	0	172.6	8,332.952	0	59.8	8,579.165	2.4	42.8	8,586.991
200-10-3	0	172.8	7,772.788	0	60.1	7,977.889	1.2	43.5	7,998.952
200-10-4	0	173.4	8,203.755	0	62.4	8,335.974	0.3	41.1	8,297.955
200-10-5	0	171	7,228.651	0	56.8	7,350.535	2.6	38.5	7,364.402
200-15-1	0	158.2	6,928.481	0	53.3	7,149.666	4.4	36.9	7,199.991
200-15-2	0	153.8	7,491.654	1.5	46	7,592.900	9.2	37.9	7,589.134
200-15-3	0	158.7	7,543.940	0	53.5	7756.547	3.7	41.3	7,764.329
200-15-4	0	158.9	8,066.942	0	51.9	8,183.756	4	38.1	8,115.711
200-15-5	0	140.8	7,482.333	4.7	45.1	7,555.261	12.6	42.8	7,615.827
300-15-1	0	244.6	10,217.101	0	80.6	10,628.301	2.1	60.5	10,607.957
300-15-2	0	236.5	11,253.393	0.9	62.3	11,482.303	10.1	60.7	11,438.291
300-15-3	0	244.9	10,976.929	0	82.2	11,356.3691	2.9	61.9	11,376.770
300-15-4	0	237.1	11,728.895	0	78.7	11,966.410	3.5	59.2	11,997.771
300-15-5	0	238.5	11,142.213	0.8	66.9	11,292.622	9.9	58.4	11,341.493
300-20-1	0	226.4	10,903.233	0	73.4	11,231.359	4.7	60.1	11,250.948
300-20-2	0.2	212.7	11,782.898	2.9	64	12,098.070	11.5	63.5	12,136.390
300-20-3	0	227	10,236.996	0	70.6	10,545.753	6.5	61.1	10,563.364
300-20-4	0	236.1	11,543.552	0.9	65.2	11,831.730	8.4	60.8	11,858.952
300-20-5	0	222.1	10,592.643	2	61.2	10,832.422	11.5	58.3	10,869.586

From the table, we can see that LS has better performance than GA and PSO within the limitation of running time (30 s for each run). Although GA achieves a better performance when comparing the results produced by PSO, the performance of GA is inferior to the proposed local search algorithm. In fact, the local search algorithm has the best average results for all the instances we test. LS yields the solutions that can process all the tasks for all the instances except 150-15-2 and 300-20-2, and more than a half of tasks are assigned to the local machines to achieve low latency and high security. In comparison, GA has 11 instances whose rejected rate is not zero, and more tasks have to be assigned to remote machines. Moreover, PSO performs the worst among the three algorithms. Besides, LS has smaller costs compared with the values of GA. Therefore, LS performs best on all the metrics.

Similarly, Table 2 gives the best results of all 45 instances. For each instance, we pick out the best solution over 10 runs according to the number of rejected tasks and the total cost, and then list all the best solutions for the three algorithms.

**Table 2:** Comparative results of the best solution over 10 runs.

	LS			GA			PSO		
Instance	#Rejected	#Local	Cost	#Rejected	#Local	Cost	#Rejected	#Local	Cost
100-5-1	0	98	3,749.571	0	59	3,819.108	0	47	3,781.272
100-5-2	0	98	3,902.546	0	62	3,970.117	0	52	4,029.095
100-5-3	0	97	3,701.371	0	58	3,717.753	0	41	3,776.172
100-5-4	0	98	4,314.778	0	58	4,301.349	0	46	4,362.843
100-5-5	0	98	3,741.932	0	59	3,868.527	0	42	3,902.980
100-10-1	0	88	3,447.738	0	38	3,515.419	1	23	3,484.749
100-10-2	0	86	4,157.000	0	38	4,259.782	0	17	4,271.786
100-10-3	0	83	3,829.099	0	35	3,937.409	1	21	3,959.419
100-10-4	0	88	4,061.314	0	37	4,093.564	0	24	4,156.844
100-10-5	0	77	3,693.829	1	27	3,680.605	5	24	3,712.366
150-5-1	0	147	5,574.434	0	89	5,695.708	0	68	5,584.052
150-5-2	0	148	5,865.309	0	83	6,009.060	0	71	6,146.270
150-5-3	0	147	5,694.042	0	85	5,919.540	0	67	5,925.531
150-5-4	0	147	6,190.606	0	77	6,238.388	0	54	6,171.385
150-5-5	0	148	5,414.249	0	83	5,594.633	0	66	5,604.121
150-10-1	0	134	5,294.551	0	53	5,376.953	1	28	5,436.064
150-10-2	0	128	6,241.442	0	51	6,421.258	1	36	6,344.115
150-10-3	0	131	5,641.550	0	55	5,880.465	0	28	5,835.448
150-10-4	0	135	6,288.819	0	53	6,338.471	1	37	6,269.320
150-10-5	0	136	5,349.565	0	51	5,430.901	1	33	5,481.943
150-15-1	0	115	5,049.656	0	48	5,146.934	4	26	5,155.840
150-15-2	0	103	5,567.713	3	38	5,721.069	9	27	5,670.857
150-15-3	0	123	5,766.045	0	45	5,826.367	1	32	5,880.845
150-15-4	0	119	6,187.288	0	24	6,197.557	7	32	6,211.291
150-15-5	0	118	5,779.109	1	40	5,807.097	6	35	5,868.481
200-10-1	0	177	6,892.485	0	66	7,051.450	0	53	7,099.090
200-10-2	0	177	8,395.566	0	65	8,548.144	1	39	8,590.509
200-10-3	0	176	7,781.355	0	65	7,978.409	0	44	7,977.051
200-10-4	0	178	8,147.028	0	66	8,284.278	0	49	8,226.066
200-10-5	0	177	7,214.994	0	62	7,402.267	1	40	7,344.745
200-15-1	0	168	6,871.216	0	57	7,141.375	1	45	7,160.826
200-15-2	0	159	7,508.692	1	49	7,615.225	6	31	7,584.174
200-15-3	0	163	7,544.616	0	58	7,692.403	1	40	7,816.401
200-15-4	0	164	8,047.307	0	56	8,191.826	1	36	8,190.958
200-15-5	0	150	7,443.951	3	43	7,571.427	10	46	7,591.578
300-15-1	0	251	10,226.541	0	84	10,599.136	1	69	10,618.304
300-15-2	0	248	11,324.833	0	57	11,520.125	8	60	11,367.876
300-15-3	0	252	10,968.732	0	90	11,329.991	0	56	11,378.326
300-15-4	0	241	11,688.697	0	87	11,988.589	1	58	12,183.619
300-15-5	0	246	11,144.910	0	57	11,350.479	9	68	11,273.256
300-20-1	0	237	10,882.128	0	78	11,256.136	1	69	11,153.087
300-20-2	0	228	11,766.276	2	67	12,211.728	8	64	12,131.008
300-20-3	0	232	10,235.831	0	76	10,618.108	4	63	10,488.928
300-20-4	0	242	11,580.021	0	65	11,849.761	3	62	11,827.778
300-20-5	0	234	10,627.410	1	58	10,744.919	8	57	10,883.312

In Table 2, it is clear that our local search algorithm is still better than GA and PSO, since its best solutions are better than those of GA. Note that although GA and LS have the same number of rejected tasks for most instances, the tasks assigned to local machines are quite different. LS performs far better than GA, because it achieves the number of 155.3 task on average whereas GA has only 58.9 tasks on average. so LS has a good ability to solve the distributed problems. Besides, it is easy to see that PSO produces solutions with more rejected tasks and a larger number of tasks with non-local machines compared to LS, so it has an unsatisfactory performance for solving the problems.

### Parameter analysis

In this subsection we analyze the penalty strategy in our algorithm. In the strategy, the cost of non-local tasks are multiplied a coefficient to penalize the assignment to a non-local machine. This is a pre-defined parameter in our algorithm. It is used to ensure tasks are assigned to their local machines as many as possible.

**Figure 1:**
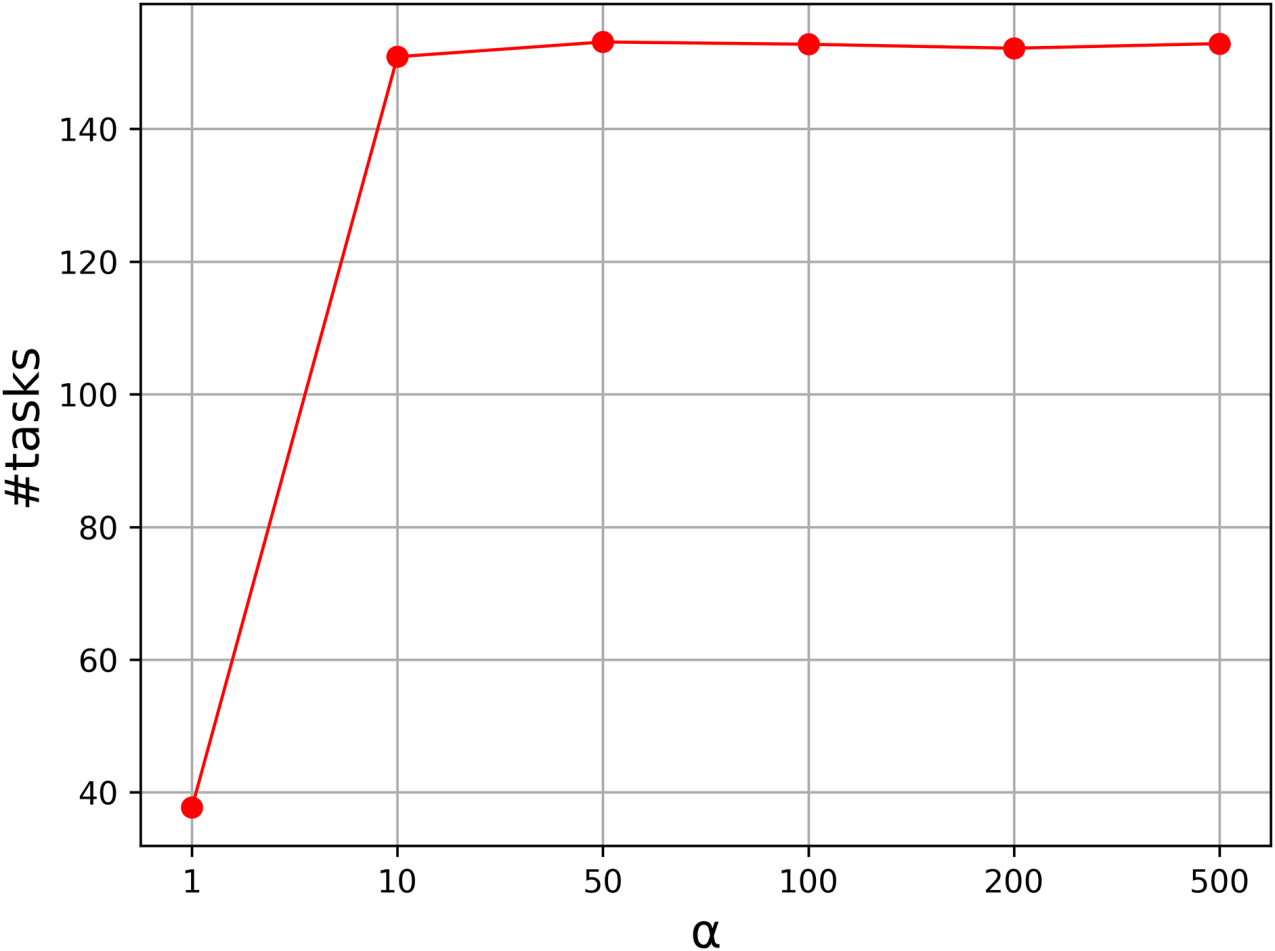
Parameter analysis on *α*.

To show the effectiveness of the strategy, we carried out a comparative experiment. In the strategy, there is a parameter to specify the level of the penalty, and it controls the weights of the penalized cost. Usually, the parameter is a real number above 1.0. To test the effectiveness of the mechanism, we tested the instances with *n* = 100 to 300, and vary the parameter *α* from 1.0 to 500, and nine values are taken into consideration. Figure 1 shows the average number of the tasks assigned to local machines with *α* = 1, 10, 50, 100, 200, 500. From the curve, we can see that the result is unsatisfactory when *α* = 1, and the average number increases greatly as *α* increases. The number becomes stable when *α* is above 50. Therefore, we set *α* to 50 in our experiment.

**Figure 2:**
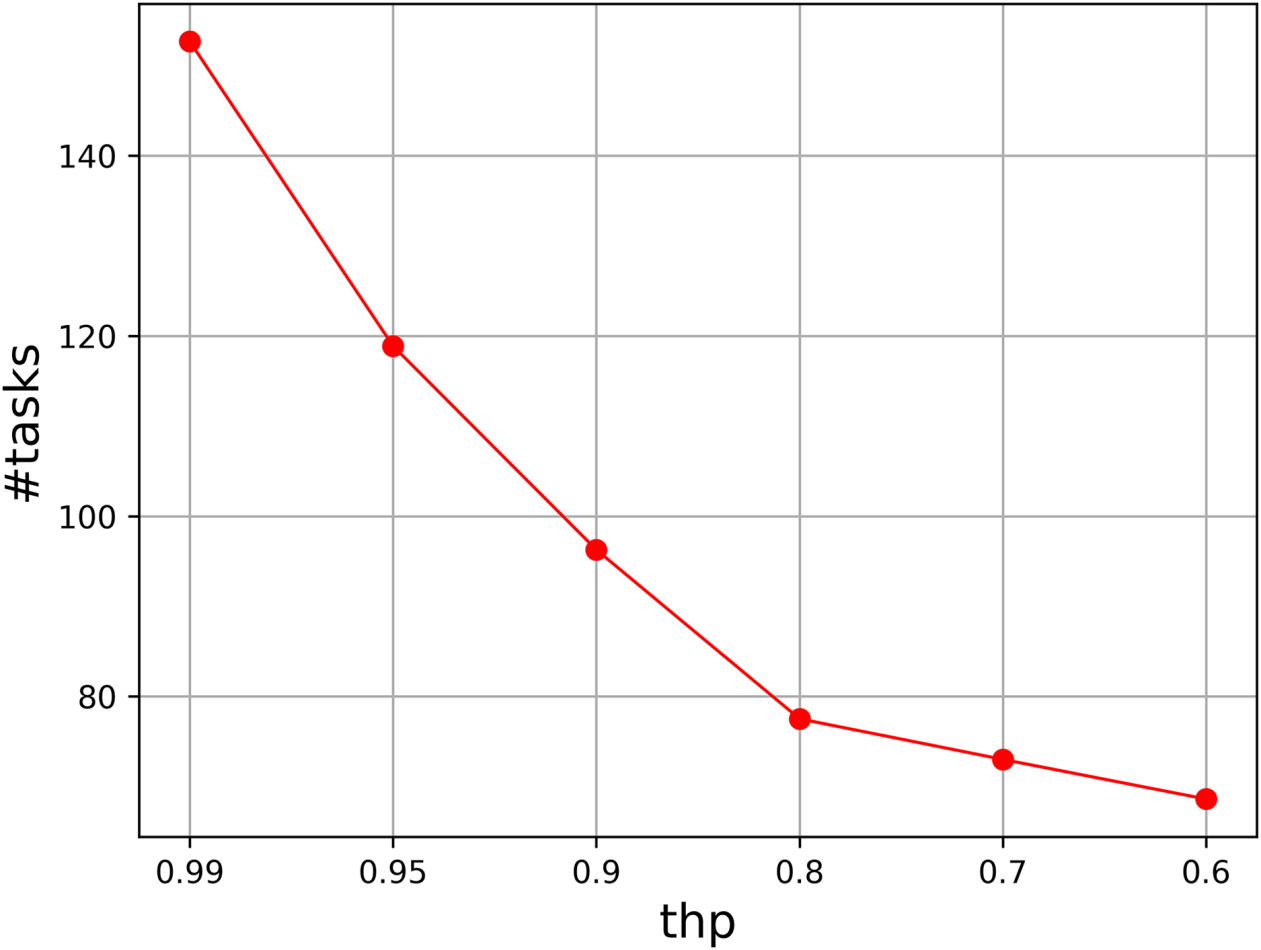
Parameter analysis on *thp*.

We also test the parameter *thp*, which determines the probability of perturbation. We vary it from 0.99 to 0.6, and select six values to show the tendency when the probability decreases. Figure 2 illustrates the results, where the accumulated value of the assigned task numbers are given in the figure. It is easy to see that *thp* = 0.99 has a fast better accumulated result than those of other values. The result becomes worse as *thp* decreases, so *thp* should be set to a probability that is close to 1.

**Figure 3:**
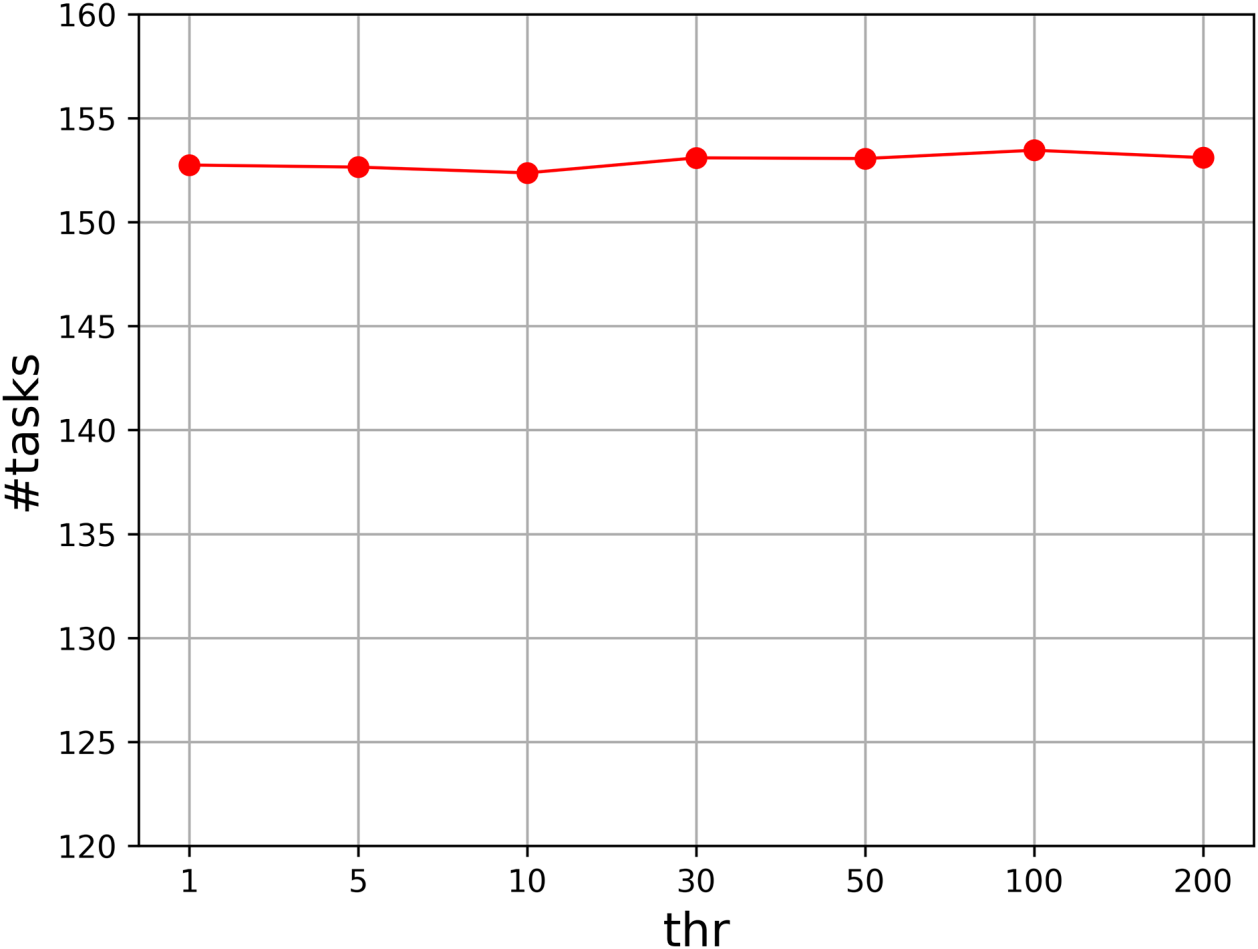
Parameter analysis on *thr*.

Moreover, we evaluate the parameter *thr* that controls the number of loops. We set *thr* to $\left\{ {1,5,10,30,50,100,200} \right\} \times n \times m$, and compute the accumulated value of the assigned task numbers. Figure 3 shows the result. As can be seen that the result for each value is quite similar to those of others, so our algorithm is not sensitive to the parameter *thr*. We set it to $5 \times n \times m$ in our experiment.

## Conclusion

Task scheduling with deadline constraints has a great importance in distributed cloud computing, which is a necessary technique for real-world applications in the smart city. In this article, we discuss task scheduling in a distributed cloud environment, where local micro cloud systems are located at different places. We also present a task scheduling problem with deadline constraints to achieve low-latency services and minimize the total cost. An efficient local search algorithm is proposed to solve the problem. The algorithm employs a greedy strategy to search for a better solution on the neighborhood and improves the solution iteratively. Moreover, randomized methods are also integrated into task selection and machine selection in order to make a better search diversity. Extensive computational experiments are performed to evaluate our proposed algorithm. The results of the experiments are compared with results produced by swarm-based approaches. Comparative analysis shows that the proposed local search algorithm performs better than existing algorithms on both the refuse rate of tasks and the total costs of the services.

## Supplemental Information

10.7717/peerj-cs.1346/supp-1 Supplemental Information 1 Code.

10.7717/peerj-cs.1346/supp-2 Supplemental Information 2 Instances.

## Additional Information and Declarations

### Data Availability

The following information was supplied regarding data availability: The raw data and code are available in the Supplemental Files.
